# Integrated Microbiome and Serum Metabolome Analysis Reveals Molecular Regulatory Mechanisms of the Average Daily Weight Gain of Yorkshire Pigs

**DOI:** 10.3390/ani14020278

**Published:** 2024-01-16

**Authors:** Shan Jiang, Jinglei Si, Jiayuan Mo, Shuai Zhang, Kuirong Chen, Jiuyu Gao, Di Xu, Lijing Bai, Ganqiu Lan, Jing Liang

**Affiliations:** 1 College of Animal Science & Technology, Guangxi University, Nanning 530004, China; js591954779@163.com (S.J.); jinglei7139@126.com (J.S.); gxu_mojiayuan@163.com (J.M.); zs714096718@163.com (S.Z.); 15737555093@163.com (K.C.); 18275790313@163.com (J.G.); xudi18788704824@163.com (D.X.); gqlan@gxu.edu.cn (G.L.); 2 Shenzhen Branch, Guangdong Laboratory of Lingnan Modern Agriculture, Key Laboratory of Livestock and Poultry Multi-Omics of MARA, Agricultural Genomics Institute at Shenzhen, Chinese Academy of Agricultural Sciences, Shenzhen 518124, China; 3 Guangxi State Farms Yongxin Animal Husbandary Group Co., Ltd., Nanning 530022, China

**Keywords:** intestinal microbiota, serum metabolites, co-occurrence network, *Lactobacillus*, *Streptomyces*

## Abstract

**Simple Summary:**

Average daily weight gain (ADG) is a dominant index for assessing the growth performance of pigs. In this study, we investigated the profound ramifications of microbiota and metabolites on the ADG of pigs. 16S rRNA sequencing and non-targeted metabolomics analysis were employed for identifying remarkably distinct microbiota and metabolites. The co-occurrence network analysis was conducted to explore the interaction between the fecal microbiota and serum metabolites. The results include ADG performance data, microbiota data, metabolite data, and the co-occurrence network, providing a valuable reference for the identification of molecular markers associated with ADG in the swine industry.

**Abstract:**

The average daily weight gain (ADG) is considered a crucial indicator for assessing growth rates in the swine industry. Therefore, investigating the gastrointestinal microbiota and serum metabolites influencing the ADG in pigs is pivotal for swine breed selection. This study involved the inclusion of 350 purebred Yorkshire pigs (age: 90 ± 2 days; body weight: 41.20 ± 4.60 kg). Concurrently, serum and fecal samples were collected during initial measurements of blood and serum indices. The pigs were categorized based on their ADG, with 27 male pigs divided into high-ADG (HADG) and low-ADG (LADG) groups based on their phenotype values. There were 12 pigs in LADG and 15 pigs in HADG. Feces and serum samples were collected on the 90th day. Microbiome and non-targeted metabolomics analyses were conducted using 16S rRNA sequencing and liquid chromatography-mass spectrometry (LC-MS). Pearson correlation, with Benjamini–Hochberg (BH) adjustment, was employed to assess the associations between these variables. The abundance of *Lactobacillus* and *Prevotella* in LADG was significantly higher than in HADG, while *Erysipelothrix*, *Streptomyces*, *Dubosiella*, *Parolsenella*, and *Adlercreutzia* in LADG were significantly lower than in HADG. The concentration of glutamine, etiocholanolone glucuronide, and retinoyl beta-glucuronide in LADG was significantly higher than in HADG, while arachidonic acid, allocholic acid, oleic acid, phenylalanine, and methyltestosterone in LADG were significantly lower than in HADG. The *Lactobacillus*–*Streptomyces* networks (*Lactobacillus*, *Streptomyces*, methyltestosterone, phenylalanine, oleic acid, arachidonic acid, glutamine, 3-ketosphingosine, L-octanoylcarnitine, camylofin, 4-guanidinobutyrate 3-methylcyclopentadecanone) were identified as the most influential at regulating swine weight gain. These findings suggest that the gastrointestinal tract regulates the daily weight gain of pigs through the network of *Lactobacillus* and *Streptomyces*. However, this study was limited to fecal and serum samples from growing and fattening boars. A comprehensive consideration of factors affecting the daily weight gain in pig production, including gender, parity, season, and breed, is warranted.

## 1. Introduction

As a pivotal trait within the livestock industry, the average daily weight gain (ADG) is intricately tied to economic benefits. It represents the growth rate of market animals and signifies the time required to reach the target weight [1]. The calculation of ADG requires measures of body weight or estimated morphometric measurement data [2]. Nutrition, genetics, environment, health, and other factors are the main factors affecting animal ADG. Different breeds may exhibit distinct abilities in nutrient absorption due to their unique intestinal microbiota, influencing feed efficiency and daily weight gain [3].

The gut microbiota significantly contributes to both the health and production efficiency of pigs. A myriad of microorganisms inhabits the intestinal mucosa of swine, playing a pivotal role not only in host metabolism and immunity but also in influencing behavioral patterns [4,5]. In goats, the abundance of *Ruminococcus* in the rumen exhibits a positive correlation with ADG [6]. *Oscillospirales*, recognized as butyrate producers [7], demonstrate a positive correlation with an increase in body weight [8]. The presence of the *Prevotellaceae_NK3B31_group* in the gut bacterial community is associated with elevated levels of acetic acid, butyric acid, and propionic acid [9]. *Alistipes* primarily produces succinic acid, with smaller amounts of acetic acid and propionic acid [10]. Screening molecular markers contributing to phenotypic variations from serum metabolite levels is a feasible approach [11]. Among short-chain fatty acids (SCFAs), only propionic acid exhibits a statistically significant association with ADG (R = 0.45, *p* < 0.05) [6]. In broilers, the *Prevotellaceae_NK3B31_group* and *Alistipes* have been robustly demonstrated to exhibit a substantial association with an elevation in ADG [12]. Antecedent research emphatically highlights the established efficacy of postbiotics derived from *Lactobacillus plantarum*, demonstrating their proven capacity to enhance ADG while concurrently diminishing the feed conversion ratio (F/G) [13]. A robust positive correlation emerged between ADG and the relative abundance of *F082*, *Saccharimonas*, and *Streptococcaceae* [14]. This underscores the pivotal role of the gut microbiota in unraveling the intricate regulatory mechanism of ADG.

Metabolomics, an innovative omics technology, has gained prominence both domestically and internationally. Essentially, ADG exhibits a noteworthy negative correlation with the relative abundance of butyrivibrio and saccharofermentans. (*p* < 0.05) [6]. Simultaneously, ADG exhibits a substantial positive correlation with the concentrations of total essential amino acids (His, Trp, Arg, Val, Gly, and Ala) in rumen fluid (r  >  0.2, *p* < 0.05) [14]. The enzyme activities of complexes (I, IV, and V) in cows manifested a compelling correlation with ADG [15]. Research on cattle has illuminated how an elevation in serum branched-chain amino acids (BCAAs) intricately links to the amplified growth rate and has been substantiated [16]. In contrast, P-salicylic acid, deoxycholic acid 3-glucuronide, glycocholic acid, and aerobactin showed a negative correlation with ADG [17]. Despite the identification of specific alterations in serum metabolites, conspicuous discrepancies exist between the outcomes observed in human subjects and animal models.

A robust correlation exists between the gut microbiota and serum metabolites. Using 16S rRNA sequencing and serum metabolomics, *Oscillibacter* demonstrated a positive correlation with chenodeoxycholic acid, glycocholic acid, and deoxycholic acid [18]. The glucose and lipid metabolic pathways in the host can be modulated using SCFAs originating from microbial sources, and SCFAs have the capacity to exert an influence on immune function and inflammation [19]. At the species level, a positive association was observed between *Bacteroides cellulosilyticus* and L-glutamine, while *B. ovatus* and *B. cellulosilyticus* exhibited a negative association with L-glutamate [20]. Nevertheless, comprehensive research is scarce on the utilization of 16S rRNA sequencing technology in conjunction with serum metabolomics, specifically in the context of ADG in pigs.

The present study focused on male growing-finishing pigs as the experimental subjects, employing 16S rRNA sequencing technology to assess bacterial abundance in the microbiota. Additionally, cutting-edge technology was leveraged to analyze the composition of metabolites in serum samples derived from the swine. The application of 16S rRNA sequencing and metabolomic technology enabled the screening of key biological molecular markers modulating ADG in pigs. We endeavor to identify metabolites and microorganisms intricately linked to ADG. From the perspective of molecular nutrition, these findings can be applied to improve the rearing environment in the swine industry, regulating weight and optimizing economic profits.

## 2. Materials and Methods

### 2.1. Animals and Sample Collection

A cohort of 350 purebred Yorkshire pigs (150 boars, 200 sows) was reared on a farm in Nanning, Guangxi, China. The piglets were weaned at 28 days of age and subsequently reared in common nursery environments. Upon reaching 70 days of age, all pigs involved in the research were transferred to a state-of-the-art fattening house. During the testing phase, each pig was fitted with a unique electronic tag on its ear, and the production performance was assessed 10 days after the adaptation period (at 80 days of age). The experimental growth data of pigs during the fattening period, specifically from day 90 to day 160, were collected using the Osborne Feed Intake Recording Equipment (FIRE) system (version 2.2.0.6, Kansas, America). Phenotypic data outliers were eliminated using Excel (version Microsoft Excel 2016), involving a normal distribution test and descriptive statistical analysis. Through the analysis of growth data, we calculated the ADG of experimental pigs using the formula ADG = (100 kg−30 kg)/required days. The pigs were provided with unrestricted access to feed, and their diet consisted of the same basal components. Fecal and serum samples were collected at the age of 80 ± 1.15 days. Following collection, fecal samples were swiftly conserved in liquid nitrogen for snap-freezing, ensuring preservation at −80 °C within the laboratory. The serum was meticulously isolated through centrifugation at 2000 rpm for 15 min, maintaining a temperature of 4 °C, and subsequently preserved at −80 °C until use. ADG was used to measure growth traits. Twenty-seven pigs with extreme phenotypes were categorized into the following two groups: LADG with 12 pigs and HADG with 15 pigs.

### 2.2. Amplicon Sequencing of 16S rRNA and Subsequent Analysis

Microbial genomic DNA was isolated by a reputable commercial provider (Shanghai Majorbio Bio-Pharm Technology Co, Ltd., Shanghai, China). The 16S rRNA gene region spanned from the V3–V4 region (extracted DNA using the primers 341F-806R, Wuhan Jinkairui Biotechnology Co., Ltd., Wuhan, China). The samples were subjected to sequencing on the Illumina MiSeq platform (Illumina, San Diego, CA, USA). Subsequent research on the obtained gene sequences was conducted using the software QIIME 2 [21]. Additionally, amplicon bioinformatics analysis was performed utilizing EasyAmplicon v1.0 software [22]. The results were then exported for further analysis in the R environment [23].

### 2.3. Untargeted Metabolomics Study and Analysis

Untargeted metabolomics analysis was conducted by the reputable organization Metware (Wuhan, China). The serum samples were processed according to the delivery standard of the Metware company [24]. Mass spectrometers (QTOF/MS-6545 and 1290 Infinity LC, produced by AB SCIEX, Foster City, CA, USA) were utilized for the experimental analysis. Raw data underwent a conversion to the mzML format using Proteo Wizard software (version 3.0), followed by the extraction of data peak values using the XCMS program (version 3.18.0) [25]. The SIMCA-P program (version 14.1) was used to perform the analysis. A significant threshold of *p*-value ≤ 0.05 was set depending on the univariate T-test analysis. A fold change was considered significant if it exceeded 1.3 or was below 0.7 (≥1.3 or ≤0.7). The ultimate data in the current study was presented using Rstudio (version: 1.4.1717) [23].

### 2.4. Investigating the Connection between Microbiota and Metabolites Using the Co-Occurrence Network

The Pearson correlation function in R is utilized to calculate the association between distinct microbes in feces and various metabolites in the serum. Subsequently, the Benjamini–Hochberg (BH) procedure is applied to adjust the resulting *p*-values from the Pearson correlation. If it possesses an adjusted *p*-value below 0.05 and an absolute value exceeding 0.6, Cytoscape_v3.9.0 can be utilized to build the network, illustrating the relationship between the gastrointestinal microbiota and the significant serum metabolite.

## 3. Results

### 3.1. ADG Performance Analysis

A significant disparity in ADG performance was observed between the HADG and LADG groups. (Table S1) (*p* < 0.05).

### 3.2. Fecal Microbiota Signatures

**Figure 1:**
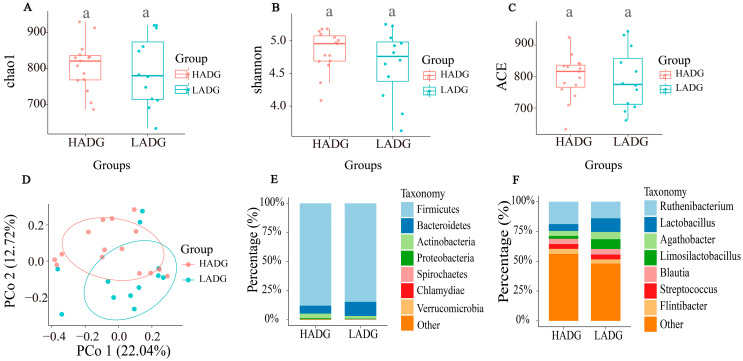
The α-diversity, β-diversity, and taxonomy between HADG and LADG. (**A**) The chao1 index. (**B**) The Shannon index. (**C**) The ACE index. (**D**) β-diversity. (**E**) The distribution of microbiota at the phylum level. (**F**) The distribution of microbiota at the genus level. If the small letters are the same, it indicates no significant difference (*p* > 0.05).

Contrasting the gut microbiota variances between LADG and HADG through sequencing, we ensured quality control and removed duplicate sequences. In our study, a comprehensive set of 1256 amplicon sequence variants (ASVs) was identified, and 490 ASVs (39.0% of the total) were detected in every single sample. The α-diversity index (Shannon, ACE, and Chao 1) showed no significant difference between HADG and LADG (Figure 1A–C). β-diversity analysis revealed partial differences between LADG and HADG. PCoA1 and PCoA2 accounted for 22.04% and 12.72% of the observed variation (Figure 1D).

The taxonomy was depicted at both the phylum level (Figure 1E) and the genus level (Figure 1F). *Firmicutes* and *Bacteroidetes* were identified as the predominant bacteria at the phylum level, collectively comprising more than 95.4% of the total. Specifically, *Firmicutes* exhibited an average abundance of 86.28%, while *Bacteroidetes* accounted for 9.18% and *Actinobacteria* contributed 3.2%. The remaining bacteria, including *Proteobacteria*, *Spirochaetes*, *Verrucomicrobia*, *Chlamydiae*, and others, collectively constitute less than 1% of the total abundance. In our analysis of the microbial composition, a total of 186 genera were detected. Notably, *Blautia* (4.48%), *Agathobacter* (5.04%), *Ruthenibacterium* (16.7%), *Lactobacillus* (8.25%), *Limosilactobacillus* (5.04%), *Streptococcus* (4.03%), *Flintibacter* (3.90%), *Duncaniella* (3.63%), and *Prevotella* (3.52%) were predominantly detected at the genera level (Figure 1F). The relative abundance of *Lactobacillus* in the LADG was significantly higher than in the HADG.

**Figure 2:**
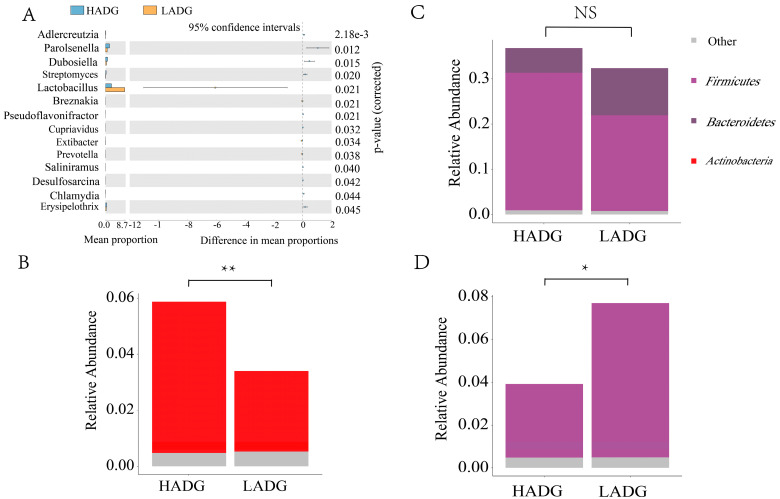
The Stamp analysis and BugBase analysis between HADG and LADG. (**A**) Microbiota with significantly different abundance at the genus level. (**B**) Contribution rate in the formation of biofilm. (**C**) Contribution rate in potentially pathogenic organisms. (**D**) Contribution rate in aerobics. NS: No Significance, *: *p* < 0.05, **: *p* < 0.01.

Moreover, the genera *Prevotella* and *Lactobacillus* were noticeably higher in LADG (*p* < 0.05), while the abundance of *Streptomyces*, *Parolsenella*, and *Erysipelothrix* was noticeably elevated in the HADG (*p*< 0.05) (Figure 2A). Compared to LADG, HADG significantly reduced the abundance of aerobic bacteria and enhanced biofilm formation capacity (*p* < 0.05) (Figure 2B–D). A stronger biofilm formation capacity facilitates the shortening of tissue cell growth cycles, accelerating metabolic rates and promoting organism growth.

### 3.3. Serum Metabolic Signatures

**Figure 3:**
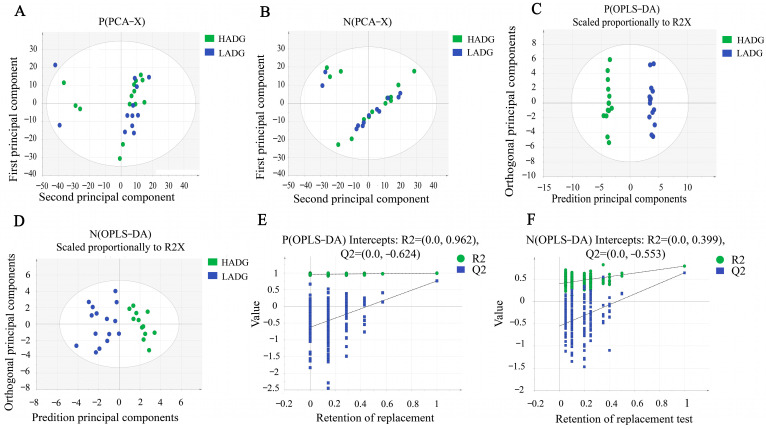
The PCA, OPLS-DA, and permutation score plots of serum samples collected from LADG and HADG based on LC-MS. (**A**) PCA score plot in the positive ion mode. (**B**) PCA score plot in the negative ion mode. (**C**) OPLS-DA score plot in the positive ion mode. (**D**) OPLS-DA score plot in the negative ion mode. (**E**) Permutation score plot in the positive ion mode. (**F**) Permutation score plot in the negative ion mode.

**Figure 4:**
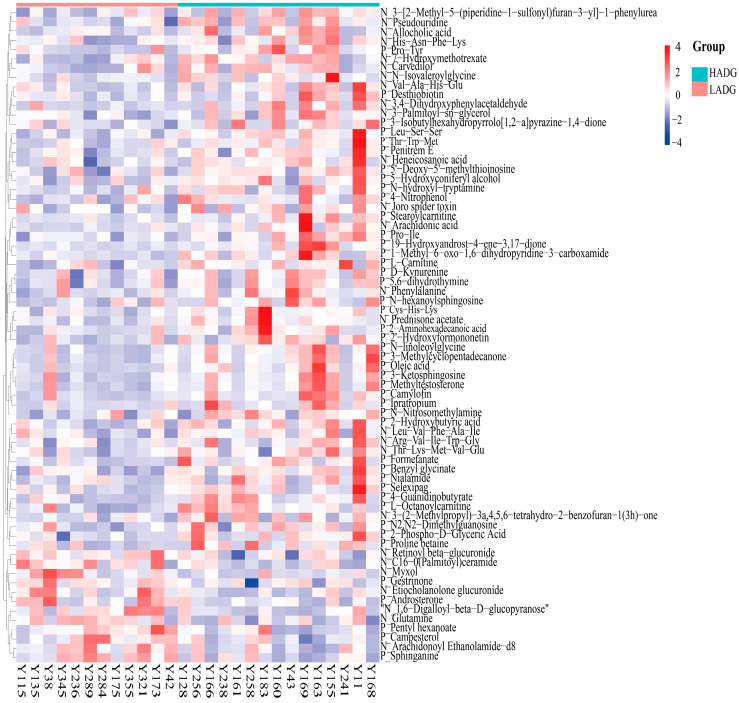
The heatmap of differential metabolites between LADG and HADG. The labels “P” and “N” denote positive and negative ion modes, respectively.

**Figure 5:**
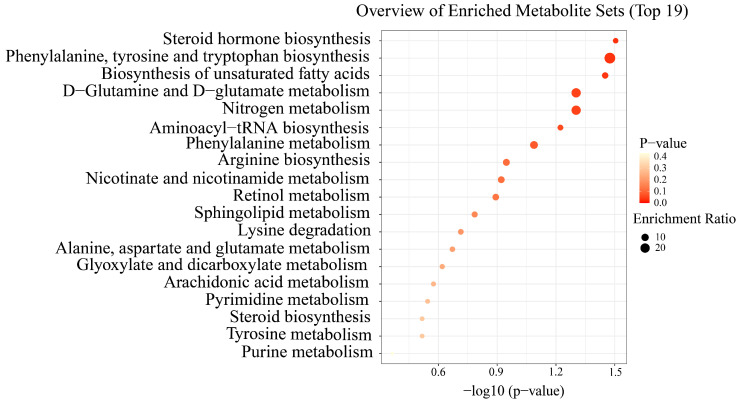
The KEGG pathways displayed enrichment in differential metabolites between LADG and HADG.

We investigated significant variations in metabolites between LADG and HADG, using untargeted metabolomics analysis on identical serum samples. Following filtration, a total of 1811 metabolites were identified in the positive ion mode, while 1039 metabolites were detected in the negative ion mode. The values of PCA analyses for the mode interpretation rates of X (R2X) in both modes exceeded 36.67% (Figure 3A,B). The OPLS-DA analysis in the positive ion mode and the negative ion mode alongside the R-squared values (R2Y) for mode interpretation were 0.98 and 0.79 (Figure 3C,D), and the prediction ability (Q2) was 0.762 and 0.642, respectively. The intercept of the permutation test was found to be lower than −0.55 (Figure 3E,F). In the comparative analysis between LADG and HADG, a comprehensive spectrum of 18 distinct metabolites was discerned in the negative ion mode (Table S2), while a distinct set of 52 metabolites emerged in the positive ion mode (Table S3). Distinct metabolites were acquired in the two modes, respectively (Figure 4). A total of 70 differential metabolites were found to be enriched in 19 metabolic pathways when comparing LADG to HADG (Figure 5), including steroid hormone biosynthesis, biosynthesis of unsaturated fatty acids, phenylalanine metabolism, and arachidonic acid metabolism. Notably, HADG exhibited higher enrichment in the metabolic pathways of unsaturated fatty acids (including arachidonic acid) and phenylalanine.

### 3.4. Constructing Co-Occurrence Network Unveiled the Interplay between Fecal Microbiota and Serum Metabolites

**Figure 6:**
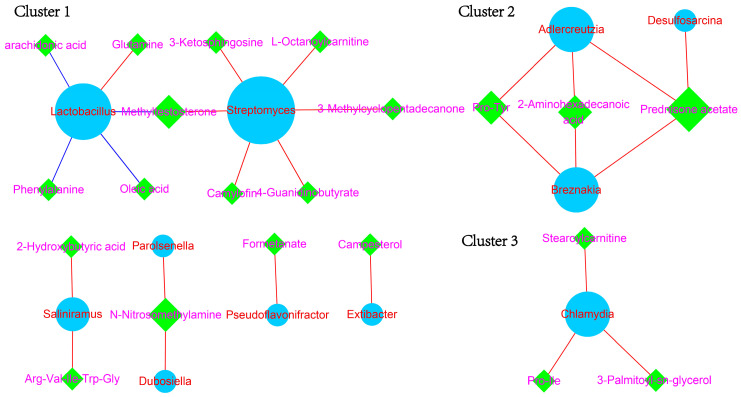
The *co-occurrence* network of fecal microbiota and serum metabolites.

The Pearson correlation coefficients were calculated using 14 differential microbiota and 70 differential metabolites. There are a total of 27 pairs of microbiota–metabolite interactions (Figure 6). Cluster one, identified in the comparison between LADG and HADG, consisted of *Lactobacillus*, *Streptomyces*, oleicacid, methyltestosterone, phenylalanine, arachidonic acid, 3-methylcyclopentadecanone, L-octanoylcarnitine, 3-ketosphingosine, 4-guanidinobutyrate, camylofin, and glutamine. Cluster two, observed in the comparison between LADG and HADG, comprised *Adlercreutzia*, Pro-Tyr, 2-aminohexadecanoic acid, *Breznakia*, prednisone acetate, and *Desulfosarcina*. Cluster three, found in the comparison between LADG and HADG, included stearoylcarnitine, *Chlamydia*, Pro-Ile, and 3-palmitoyl-sn-glycerol.

## 4. Discussion

ADG, as a vital indicator of the livestock growth rate, is instrumental at enhancing economic profitability by increasing the daily weight gain of swine. In our study, we meticulously selected 350 pigs based on the ADG index, ultimately choosing 27 boars. These animals were then categorized into the LADG and HADG groups, revealing the notable discrepancies between them. Employing 16S rRNA sequencing and metabolomics profiling, our investigation aimed to identify molecular markers that were significantly associated with ADG. The primary objective of this study is to unveil the major differences in the microbiota of fecal samples and serum metabolites between LADG and HADG.

Gastrointestinal (GIT) microbiota exhibited a prevalence of the phyla *Firmicutes* and *Bacteroidetes*, aligning with the findings of previous investigations [26]. Nevertheless, the present study unveiled a noteworthy increase in the prevalence of *Firmicutes* and a simultaneous decrease in the abundance of *Bacteroidetes* in HADG compared to LADG. Given that *Firmicutes* have the potential to enhance energy extraction from the diet [27], an initially demonstrated correlation between obesity and a diminished ratio of *Bacteroidetes* to *Firmicutes* in the gut microbiota exists [28]. As the growth rate of pigs increases, there is a concomitant requirement for increased energy intake from the external environment to accelerate metabolic processes. In HADG, there was a significant increase in the abundance of *Streptomyces*. Since the 1940s, *Streptomyces* have served as probiotics, and the application of *Streptomyces aureofaciens* in animals resulted in augmented weight gain; this breakthrough paved the way for identifying the antibiotic chlortetracycline [29]. Derived from the natural synthesis of *Streptomyces fradiae*, Tylosin stands as the prevailing growth promoter employed in agricultural practices [30]. In our study, *Streptomyces* play a vital role in promoting the growth of swine. Similar to prior observations [3], significant differences were observed between these two groups, with the predominant bacterial genus being *Lactobacillus* instead of *Prevotella.* The substantial presence of *Lactobacillus* in LADG demonstrated that *Lactobacillus* inhibits the weight gain of pigs, and lower levels of *Lactobacillus casei/paracasei* and *Lactobacillus plantarum* are linked to obesity [31]. A previous study demonstrated that among the *Lactobacillus* species that have been linked to obesity [32], certain strains were found to contribute to weight loss [33]; *L. acidophilus* can be recommended as a potential intervention for ameliorating obesity [34]. *Lactobacillus plantarum* exhibited an association with weight reduction in animal studies, while *Lactobacillus gasseri* showed a correlation with weight loss in both overweight people and animal models [33]. Moreover, lactic acid is produced by *Lactobacillus* spp. and is instrumental in regulating antimicrobial, antiviral, and immune responses [35]. Pathogenic bacteria can be reduced by the substantial presence of *Lactobacillus* spp. in the gastrointestinal tract of animals [36]. There is a significant enrichment of the *Prevotella* genus in the LADG, indicating that *Prevotella* significantly decreases the daily weight gain in pigs. Undoubtedly, an abundance of *Prevotella* in the gut microbiome has been shown to enhance weight loss [37], reduce cholesterol levels [38] and restrict the bifidogenic effect [39]. It is worth mentioning that *Prevotella*, especially *P. copri*, has been observed to be associated with diet or disease [40]. Another study has shown an association between *P. copri* and insulin resistance [41]. Thus, pigs gain weight quickly by improving the abundance of *Streptomyces* and decreasing the abundance of *Lactobacillus* and *Prevotella*.

OPLS-DA metabolomics analysis revealed significant alterations in the metabolites between LADG and HADG, suggesting the trustworthiness and consistency of the OPLS-DA mode without susceptibility to overfitting. The Q2 value for both LADG and HADG exceeded 0.5. We identified 70 metabolites, including 14 amino acids and derivatives (benzyl glycinate, Pro-Tyr, Val-Ala-His-Glu, Thr-Trp-Met, Thr-Lys-Met-Val-Glu, Pro-Ile, Leu-Val-Phe-Ala-Ile, Cys-His-Lys, Arg-Val-Ile-Trp-Gly, Arg-Ile-His, phenylalanine, glutamine, N-isovaleroylglycine, His-Asn-Phe-Ly), four fatty acids (heneicosanoic acid, oleic acid, desthiobiotin, arachidonic acid), three fatty acid esters (pentyl hexanoate, stearoylcarnitine, L-octanoylcarnitine), four steroids (androsterone, 19-hydroxyandrost-4-ene-3, 17-dione, campesterol, gestrinone), one bile acid (allocholic acid), one androgen and derivatives (methyltestosterone). Differences in metabolites were observed between LADG and HADG. The arachidonic acid metabolic process indicates a compelling connection between lipid metabolism and the immune system, impacting cardiovascular and metabolic disorders [42]. The onset and resolution of inflammation are predominantly regulated by localized chemical autacoids, comprising a diverse range of proteins, peptides, and lipid-derived mediators (especially arachidonic acid-derived leukotrienes and prostaglandins) [43]. Allocholic acid, a potent of insulin secretion stimulator, exhibits varying effects on insulin resistance. PGE2, a metabolite of arachidonic acid, promotes adipogenesis, glycogenolysis, and gluconeogenesis, ameliorating insulin resistance in adipocytes [44]. The observed resistance to lipoapoptosis associated with oleic acid cosupplementation is correlated with an enhanced ability to accumulate neutral lipids. The presence of unsaturated fatty acids, whether derived from endogenous or exogenous sources, promotes the accumulation of triglycerides [45]. Treatment with oleic acid improved the inflammatory response induced by palmitic acid, leading to a reduction in cellular ROS production. ROS was found to be a potent molecule that upregulated the protein expression of JUN, and elevated ROS generation and JUN protein expression significantly hindered the insulin signaling pathway [46]. Thus, oleic acid significantly modulates the glucose metabolism pathway, facilitating increased energy intake and promoting weight gain in the organism. Methyltestosterone enhances muscle mass and strength. The consequent enhancement of physical performance triggers the activation of bone-building locations and the stimulation of bone formation-regulating cells [47]. In our study, with the accelerated weight gain rate in pigs, there is an augmented demand for denser bone tissue, providing structural support to the organism. Phenylalanine has also been linked to the risk of diabetes [48]. As blood sugar levels increased, the levels of six amino acids (including phenylalanine) also increased, while the levels of histidine and glutamine decreased. These associations between amino acids and blood sugar levels are largely attributed to changes in insulin sensitivity [49]. In this study, compared to the LADG, the HADG exhibited higher levels of phenylalanine enrichment, while the content of glutamine significantly decreased, consistent with previous findings. Rapid weight gain necessitates the expenditure of additional energy for sustenance, with the majority of energy metabolism derived from carbohydrate metabolism.

The microbiota exhibited distinguished amino acid metabolic pathways for histidine, leucine, isoleucine, and valine. Additionally, metabolite analysis revealed enrichment of pathways associated with phenylalanine, tyrosine and tryptophan, arginine, lysine, alanine, and tyrosine metabolism. In both human and animal models with evident obesity, a distinct expression pattern of plasma amino acid levels [50], particularly an elevation in BCAAs, notably contrasted with the control group. The introduction of valine resulted in independent and significant increases in litter weaning weights [51]. Metabolomics research has shown a correlation between the concentration of BCAAs in the blood and insulin resistance with weight loss [52]. The microbiota in HADG, compared with LADG, exhibited significant enrichment in energy metabolism, insulin resistance, and insulin signaling pathways, with a curbed purine metabolism. The biosynthesis of unsaturated fatty acids, steroid biosynthesis, arachidonic acid metabolism, and pyrimidine metabolism pathways are enriched in metabolites. Purine metabolism plays a pivotal role in the initial stages of development. Prior research predicted a close correlation between the growth rate and purine contents in slow-growing chickens [53]. Augmenting energy intake results in an increase in body weight [54]. As weight gain accelerates, there is an increase in the intensity of physiological processes within the body, demanding stronger energy intake [55]. Further data substantiate the notion that insulin sensitization is linked to the enhanced regulation of body weight [56]. It is hypothesized that this effect is instigated through the action of SCFAs on the energy storage capacity and the body’s ability to respond to energy intake via various mechanisms, including the production of anorexic hormones, the augmentation of energy expenditure, and optimization of metabolic functions in peripheral tissues, such as skeletal muscle and adipose tissue [57].

Central microbiota in the co-occurrence network were *Lactobacillus*, *Streptomyces*, and *Chlamydia*, while prednisone acetate emerged as the core metabolite. Clusters one to three observed in the comparison between LADG and HADG consisted of eleven, seven, and three pairs. The abundance of *Chlamydia* and *Streptomyces* in HADG was significantly higher than in LADG. Notably, *Lactobacillus* and *Streptomyces* were the prevalent microbiota. Clusters one to three contributed to explaining the interaction between the gut microbiome and serum metabolites in weight gain.

However, it is crucial to recognize and address the limitations inherent in our study. Firstly, we only utilized male pigs as the influence of seasonality and parity on weight changes in Yorkshire were not included in our study. Additionally, we did not include ileal samples and immunological parameters in our analysis. Despite these limitations, the findings of our study hold potential significance for the genetic improvement of the pig industry. Therefore, in future studies, we aim to consider factors such as gender, seasonality, parity, and immunological parameters and the inclusion of ileal samples to provide a comprehensive understanding of the effects that microbiotas and metabolites have on body weight change.

## 5. Conclusions

In summary, this study investigated variations in microbiota composition, metabolite levels, and the correlation between gastrointestinal microbiota and serum metabolites. The aim was to explore potential biomolecular markers influencing the weight gain of Yorkshire pigs. LADG increased the abundance of *Lactobacillus* and *Prevotella*, along with the concentration of glutamine. Conversely, HADG showed an increased abundance of *Streptomyces* and concentrations of arachidonic acid, allocholic acid, oleic acid, phenylalanine, and methyltestosterone. The networks of *Lactobacillus* and *Streptomyces* significantly impacted the regulation of pig weight gain. However, it is important to consider additional factors such as gender, parity, season, and breeds in further research. This comprehensive approach could help elucidate the mechanisms underlying changes in swine weight gain.

## Data Availability

The datasets used and/or analyzed in the current study are available from the corresponding author on reasonable request.

## Supplementary Materials

The following supporting information can be downloaded at: https://www.mdpi.com/article/10.3390/ani14020278/s1, The online version of this publication includes supplementary material for further reference. Additional File S1: Table S1, The ADG performance analysis between LADG and HADG. Table S2, Details of distinct metabolites in the negative ion mode between LADG and HADG. Table S3, Details of differential metabolites in the positive ion mode between LADG and HADG.
